# Nanog and β-catenin: A new convergence point in EpSC proliferation and differentiation

**DOI:** 10.3892/ijmm.2011.871

**Published:** 2011-12-29

**Authors:** DELONG YIN, LINQIANG TIAN, YAPING YE, KUNPENG LI, JIANG WANG, PENG CHENG, ANMIN CHEN, FENGJING GUO, HUI HUANG

**Affiliations:** Department of Orthopedics, Tongji Hospital, Tongji Medical College, Huazhong University of Science and Technology, Wuhan, Hubei 430030, P.R. China

**Keywords:** Nanog, Wnt/β-catenin, epidermal stem cells

## Abstract

Skin tissue homeostasis is maintained by the balanced proliferation and differentiation of certain types of proliferating cells such as epidermal stem cells (EpSCs). The proliferation and differentiation of EpSCs are complex processes which are not well understood. This study aimed to find the internal relationship between the Nanog pathway and the Wnt/β-catenin pathway in the proliferation and differentiation process of EpSCs. In brief, EpSCs were isolated from rat epidermis and cultured. The MTT assay, western blotting, polymerase chain reaction (PCR) and immunocytochemistry were performed during the proliferation and differentiation process of EpSCs. Our results showed that 10^−7^ M neuropeptide substance P could effectively stimulate proliferation of EpSCs and that a possible link exists between the Nanog pathway and the Wnt/β-catenin pathway.

## Introduction

Skin tissue homeostasis is maintained by two kinds of progenitor cells: epidermal stem cells (EpSCs) and their progeny, known as the transit amplifying (TA) cells (1). EpSCs are responsible for replacing the differentiated cells of the interfollicular epidermis, hair follicles and sebaceous glands (2). While little is known about the proliferation, differentiation and developmental processes of EpSCs, these processes may be regulated by a program of differential genes or different signaling pathways.

The Nanog gene is thought to be involved in the differentiation and proliferation processes of different stem cells (3–5). It was reported that the Nanog gene plays a critical role in regulating the cell fate of the pluripotent inner cell mass (ICM) during embryonic development, maintaining the pluripotent epiblast and preventing differentiation (6). The canonical Wnt/β-catenin pathway is also involved in the control of gene expression, cell behavior, cell adhesion, and cell polarity of different types of cells (7). β-catenin plays a central role in the Wnt signalling pathway, and components of the β-catenin signaling pathway are often regulated by other signals. Thus it was of great interest to study the relationship between the Wnt/β-catenin pathway and other signaling pathways (8).

In this study, we aimed to evaluate the relationship between β-catenin expression and Nanog expression. In brief, EpSCs were isolated and identified by immunofluorescence. Cells were then induced to be proliferated and differentiated. Western blotting and real-time polymerase chain reaction (PCR) were respectively performed during the proliferation and differentiation periods of EpSCs. Our results showed that 10^−7^ M neuropeptide substance P (10^−7^ M SP) could effectively induce proliferation of EpSCs. Western blotting results showed that expressions of β-catenin and Nanog both increased with culture time during the proliferation period, and gained a robust increase 48 h after the proliferation. The expression of β-catenin increased steadily with culture time during the differentiation period, and expression of the Nanog gene increased during the first 24 h of cell culture but began to decrease after the initial periods. PCR results were generally in accordance with the western blotting results. Our results demonstrated the involvement of Nanog and Wnt/β-catenin in the proliferation and differentiation process of EpSCs and suggest the existence of a potential link between these two signaling pathways.

## Materials and methods

### Cell culture

A skin tissue was obtained from the back of neonatal SD mice by plastic surgical procedures, washed in phosphate-buffered solution (PBS), and connective tissue and subcutaneous fat were removed. The skin sample was sterilized with 70% ethanol, rinsed in PBS, and minced into 5-mm wide strips using a sharp scalpel; the strips were treated with 0.25% Dispase (Roche Co., Switzerland) solution at 4°C overnight. The epidermis was mechanically separated from the dermis, and incubated in a solution of 0.25% trypsin at 37°C for 30 min to dissociate the cells; enzyme activity was then blocked with Dulbecco’s modified Eagle’s medium (DMEM; Gibco Co., USA) containing 10% fetal bovine serum (FBS; Gibco Co.) and the cells were suspended with a pipette. The cell suspension was filtered through a stainless steel mesh attached to a 60-mm cell culture plate to remove any remaining tissue pieces, and the cells were transferred to a 15-ml centrifuge tube and collected by centrifugation for 5 min at 1,000 rpm. To select stem cells, 1×10^6^ dissociated epidermal cells were plated onto collagen type IV (Sigma Co., USA) (100 μg/ml)-coated dishes at room temperature for 10 min. The unattached cells were removed, and the rapidly adherent epidermal cells were cultured with keratinocyte serum-free medium (K-SFM; Gibco Co.) supplemented with epidermal growth factor (EGF) (K-SFM; Gibco Co.) and bovine pituitary extract (BPE; Gibco Co.) and with 0.05 mM calcium chloride (CaCl_2_, Sigma Co.) at 37°C, 5% CO_2_ in a humidified incubator for two days before replacing the medium. The medium was changed every other day.

### EpSCs proliferation

EpSCs were seeded in a 35-mm culture plate treated with collagen type IV at a density of approximately 1×10^4^ cells/plate for 24 h. Cells were then induced to proliferate by adding 10^−7^ M SP (Sigma Co., USA) into the K-SFM. After treatment with 10^−7^ M SP, the cells were examined independently at three time points. The control cells were cultured in the K-SFM without SP.

### EpSCs differentiation

EpSCs were seeded in a 35-mm culture plate treated with collagen type IV at a density of approximately 1×10^4^ cells/plate for 24 h, and EpSCs were induced to differentiate by culturing in Dulbecco’s modified Eagle’s medium (DMEM; Gibco Co.) containing 10% fetal bovine serum (FBS; Gibco Co.).

### MTT assay

EpSCs were plated in 96-well plates treated with collagen type IV at a density of 2×10^4^ cells/well. Cells were then divided into two groups, the first group was the control group and the second group was exposed to 10^−7^ M SP to induce cell proliferation. Forty-eight hours post-treatment, the relative number of living cells was determined by the 3-(4,5-dimethylthiazol-2-yl)-2,5-diphenyl tetrazolium bromide (MTT) assay. Cells were incubated in the medium containing 500 μg/ml MTT (Sigma-Aldrich) for 4 h. The reaction was then terminated by incubating the cells in dimethyl sulfoxide for 10 min. The specific absorbance at 492 nm (A492) was determined. All the experiments were performed at least three times independently.

### Western blotting

Total cell lysates of EpSCs that underwent the proliferation and differentiation processes were obtained at different time points (0, 24, 48 and 72 h) by lysing the cells in RIPA buffer containing 50 mM Tris-HCl, 150 mM NaCl, 1% NP-40, 0.1% SDS, 0.5% sodium deoxycholate, 2 mM sodium fluoride, 1 mM EDTA, 1 mM EGTA and protease inhibitor cocktail. The protein concentration was determined using the bicinchoninic acid protein assay (Pierce, Rockford, IL). The proteins were separated by SDS-PAGE, transferred to nitrocellulose, blocked, and incubated with the following primary antibodies: mouse anti-β catenin (R&D Systems, USA) diluted 1:500, mouse anti-Nanog (Beijing Biosynthesis Biotechnology Co., Ltd.) diluted 1:500, and mouse anti-β-actin (Beijing Biosynthesis Biotechnology Co., Ltd.) diluted 1:100 that was used as a loading control. The membrane was washed and incubated with the respective secondary antibodies conjugated with peroxidase. Protein detection was performed with the chemiluminescence detection system (Pierce).

### PCR analysis

PCR analysis was performed 24, 48 and 72 h after cell culture. Total-RNA was extracted using the TRIzol reagent (Invitrogen, USA) according to the manufacturer’s protocol. RNA concentration and purity were determined by A260 and A260/A280 ratios, respectively. The integrity of total-RNA was assessed on standard 1% agarose/formaldehyde gels. The RNA samples were treated with DNAse I to remove residual traces of DNA. cDNA was obtained from 1 μg of total-RNA, using reverse transcriptase (Toyobo, Japan) and random primers (Promega) in a final volume of 20 μl. cDNAs (1 μl for each sample) were amplified by PCR using the primer sequences as follows: Nanog, 5′-GAGCGTGGATCTTTCTGG-3′ (sense) and 5′-TCTTGGTGAGGACCTTGTT-3′ (antisense); myc, 5′-CGCTACGTCCTTCTCCC-3′ (sense) and 5′-CCGCTC CACATACAGTCC-3′ (antisense); β-catenin, 5′-AGCAGTT CGTGGAGGGCGT-3′ (sense) and 5′-CATTCCTGGAGTGG AGCAACTCT-3′ (antisense). Thermal cycle parameters were: 95°C for 2 min, 35 cycles of 95°C for 30 sec, 52–60°C (depending on the Tm of each individual set of primers) for 1 min and 72°C for 30 sec. β-actin, 5′-AGCCATGTACGTA GCCATCC-3′ (sense) and 5′-CTCTCAGCTGTGGTGGT GAA-3′ (antisense) was amplified as an internal control. The RT-PCR products were separated by 2% agarose gel electrophoresis, stained with ethidium bromide, and photographed under UV illumination.

### Immunocytochemistry

The isolated cells were cultured in a 4-well plate at a density of approximately 1×10^4^ cells/well for 24 h. The cells were then rinsed several times with PBS, fixed with 4% paraformaldehyde solution for 15 min at room temperature, and permeated with 0.2% Triton X-100-PBS solution for 5 min. The cells were incubated with primary antibodies to anti-cytokeratin 15 antibody (CK15), anti-cytokeratin 18 antibody (CK18) (rabbit polyclonal antibody, 1:100), anti-cytokeratin 19 antibody (CK19) (rabbit polyclonal antibody, 1:100) and integrin β1 (rabbit polyclonal antibody, 1:100) (obtained from Boster Biological Technology, Ltd.) at 4°C overnight, and primary antibody binding was detected via the corresponding goat anti-rabbit IgG: FITC and goat anti-rabbit IgG Cy3 (Boster Biological Technology, Ltd.). The cells were observed under a confocal microscope (Bio-Rad, Hercules, CA, USA).

### Statistical analysis

All data are presented as the mean values ± standard deviation (SD). Statistical analyses were performed using SPSS11.0 statistics software. Differences between groups were compared using Student’s t-test. Values of P<0.05 were considered statistically significant.

## Results

### Cell morphology and immunocytochemistry

**Figure 1:**
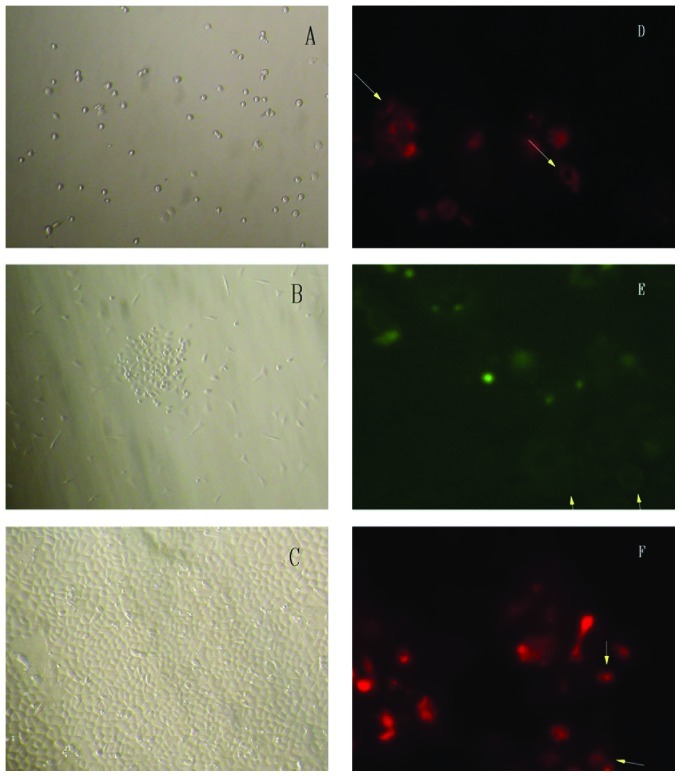
(A) The rapidly adherent EpSCs show stem cell characteristics such as a round shape, small size, few organelles. The cell formation morphologies are different, such as (B) bird nest-like, (C) slabstone-like. The expressions of CK15, CK19, and integrin β1 were examined by immunocytochemistry. Results show that (D) CK15 and (E) CK19 are highly expressed in the cytoplasm of the isolated cells but (F) integrin β1 is expressed in the nucleus of the cells.

Murine EpSCs were isolated using rapid substrate attachment. Collagen type IV was used in this study to isolate EpSCs because collagen type IV is the ligand of β1 integrin which is a potent cell marker of EpSCs. The rapidly adherent epidermal cells after 48 h of culture showed the stem cell characteristics of immaturity, round shape, small size, few organelles (Fig. 1A). Cells formed different morphologic colonies, such as bird nest-like (Fig. 1B) or slabstone-like (Fig. 1C). The expression of cytokeratin 15 (CK15), CK19, and integrin β1 was examined by immunocytochemistry. Results showed that CK15 (Fig. 1D) and CK19 (Fig. 1E) were highly expressed in the cytoplasm of the isolated cells but integrin β1 was expressed only in the nucleus of the cells (Fig. 1F). These results showed that these isolated population represented the EpSCs.

### EpSCs proliferation

**Figure 2:**
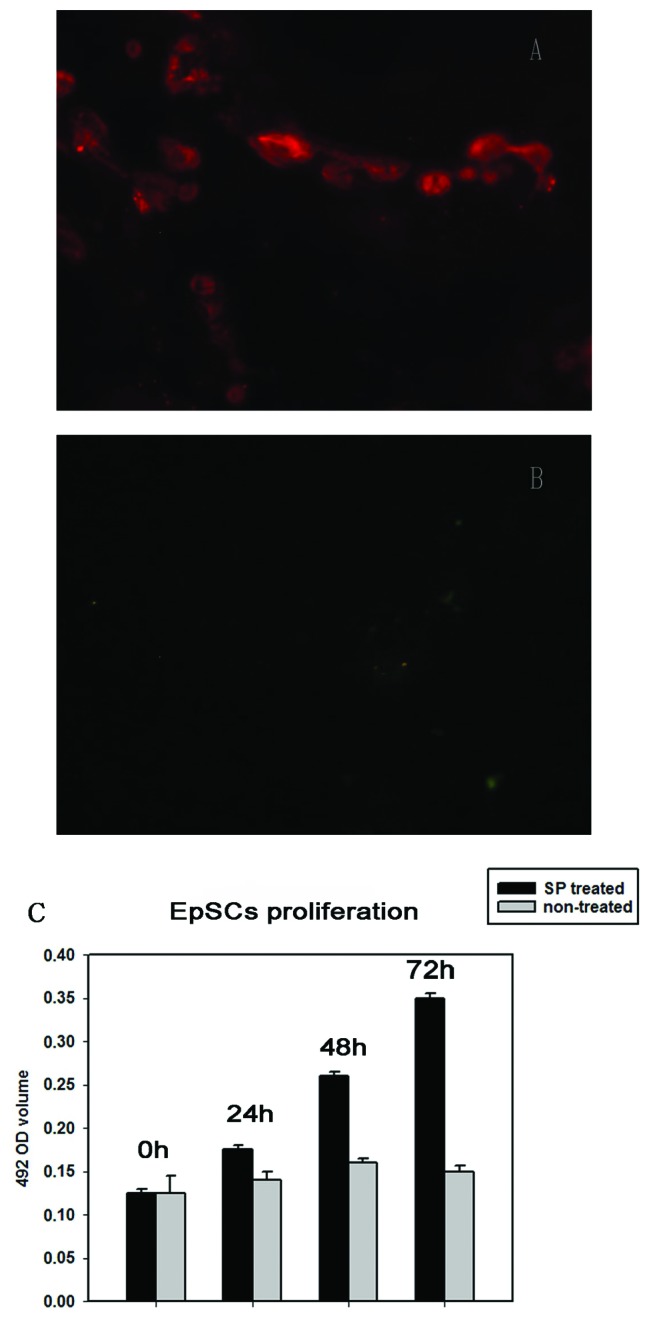
Proliferated EpSCs (A) positively express CK15 and (B) negatively express CK18 as detected by immunofluorescence. (C) The proliferative effect of 10^−7^ M SP on EpSCs was measured by MTT assay.

EpSCs were treated with 10^−7^ M SP to induce cell proliferation. The effect of 10^−7^ M SP on EpSCs proliferation was measured by the MTT assay. SP of 10^−7^ M significantly promoted the proliferation of EpSCs (Fig. 2C) and the EpSCs still preserved stemness by expressing CK15 (Fig. 2A) and not CK18 (Fig. 2B).

### Nanog and β-catenin expression profiles during the proliferation of EpSCs

**Figure 3:**
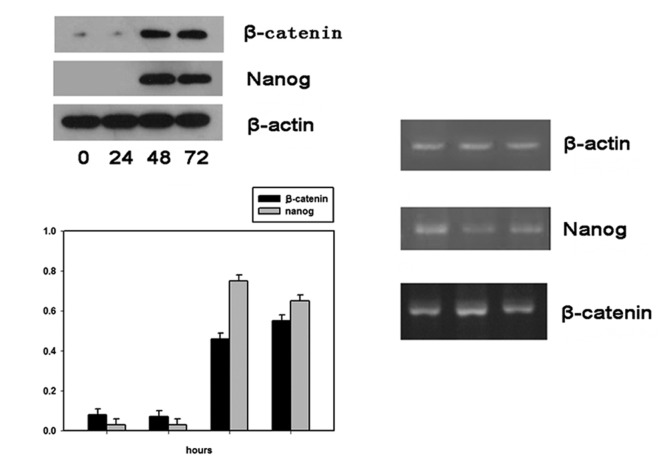
(A) EpSCs proliferation induced by 10^−7^ M SP. Western blotting results show low protein levels of both Nanog and β-catenin at the initial 24 h, but a robust increase 24 h after cell culture. (B) PCR results show that Nanog mRNA expression levels decrease with culture time while β-catenin mRNA expression levels remain high throughout the whole proliferation process.

At 0, 24, 48 and 72 h after cell culture, EpSCs of the proliferation group induced by 10^−7^ M SP were respectively analyzed for their Nanog and β-catenin expressions both at the protein and mRNA levels. Results of western blot analysis showed that protein levels of both Nanog and β-catenin were low at the initial 24 h, but robustly increased 24 h after cell culture (Fig. 3A). The PCR results showed that Nanog mRNA expression levels decreased with culture time while β-catenin mRNA expression levels remained high throughout the whole proliferation process (Fig. 3B). Thus the western blotting and PCR results suggested that both Nanog and β-catenin expression were not in accordance at the protein and mRNA levels, possibly due to asymmetric division of EpSCs.

### Nanog and β-catenin expression profiles during the differentiation of EpSCs

**Figure 4:**
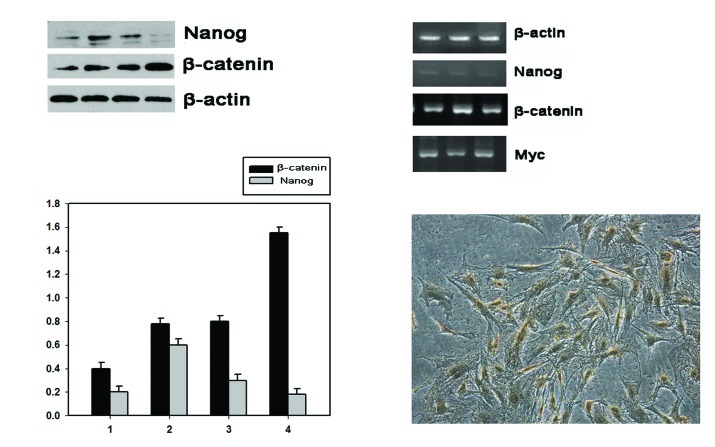
(A) EpSCs differentiation. Western blotting results show that Nanog expression increases at the initial 48 h but with a robust decrease 72 h after cell culture, while β-catenin expression levels increase with culturing time for the whole process. The PCR analysis was performed 24, 48 and 72 h after cell culture. (B) The Nanog mRNA expression levels are low for the whole process, while β-catenin and Myc mRNA expression show a stable increase for the whole cell culture process. (C) Differentiated EpSCs positively expressed CK18 as demonstrated by immunocytochemistry.

EpSCs were isolated and cultured in serum-containing culture medium DMEM containing 10% FBS to induce cell differentiation. Differentiated EpSCs positively expressed CK18 as demonstrated by immunocytochemistry (Fig. 4C). Western blotting results showed that Nanog expression increased at the initial 48 h but with a robust decrease 72 h after cell culture, while β-catenin expression levels increased with culturing time for the whole process (Fig. 4A). The PCR analysis was performed 24, 48 and 72 h after cell culture. The Nanog mRNA expression levels were low for the whole process, while β-catenin and Myc mRNA expression levels showed a stable increase for the whole cell culture period (Fig. 4B).

## Discussion

The skin tissue is of great importance for human beings. It protects internal organs from outer environmental insults, functions in body temperature regulation and participates in fluid balance (9). The epidermis is the outer layer of the skin and consists of various types of cells including stem cells such as EpSCs and TA cells (10). EpSCs is characterized by the unlimited self-renewing capacity and relatively low probability of undergoing terminal differentiation. EpSCs are of great importance in regulating human skin homeostasis. The balance between EpSCs proliferation and EpSCs differentiation is precisely controlled (11). However, the molecular mechanisms underlying these processes remain unknown.

This study aimed to elucidate the molecular mechanism that underlies EpSCs proliferation and EpSCs differentiation from the Nanog and Wnt/β-catenin perspective and tried to find out potential links between these two signaling pathways.

A majority of the isolated EpSCs positively expressed cytokeratin 15 (CK15), CK19 and integrin-β1 which suggested that cells were perfectly isolated (12–14). Then EpSCs were induced to proliferate by the addition of 10^−7^ M SP. MTT results revealed that 10^−7^ M SP promoted EpSCs proliferation 24, 48 and 72 h after cell culture. Proliferated EpSCs were then respectively tested for the CK15 and CK18 expressions by immunofluorescence. Results demonstrated that EpSCs positively expressed CK15 but negatively expressed CK18 which suggested that 10^−7^ M SP specifically promoted EpSCs proliferation but not differentiation. EpSCs were then cultured in serum-containing culture medium DMEM containing 10% FBS to induce cell differentiation. Immunocytochemistry demonstrated that a majority of EpSCs positively express CK18 which represented cell differentiation (15). EpSCs that underwent proliferation and differentiation were tested for Nanog and β-catenin expression both at the protein and the mRNA levels.

Nanog is a pluripotent state-specific transcription factor which plays a critical role in regulating cell fate during embryonic development, maintaining the pluripotent epiblast and preventing differentiation. It is also proposed as a transcription repressor that inhibits genes that are important for cell differentiation (6).

Wnt signaling is another important signaling, Wnt ligand binds to cell surface receptors, results in downstream inactivation of glycogen synthase kinase (GSK) and translocation of β-catenin to the nucleus (16). Studies have demonstrated that Wnt/β-catenin signaling activation results in generation of new hair follicles in adult epidermis (17). The effect of the Wnt signaling pathway on cell differentiation is still controversial, possibly due to the various downstream Wnt signaling pathways (18). Since β-catenin is the key effector of the Wnt signaling pathway, we thus hypothesized that there is a potential link between the Nanog pathway and the Wnt pathway through β-catenin modulation.

Results of the western blotting demonstrated that both Nanog and β-catenin were not expressed in the initial phase of cell proliferation induced by 10^−7^ M SP but showed a robust increase 48 and 72 h after cell proliferation. Results of the PCR analysis demonstrated that the Nanog mRNA expression decreased with the culture time while the β-catenin mRNA was high for the whole cell proliferation period. Thus the EpSCs both expressed Nanog protein and mRNA during the cell culture period, which indicated the multipotency or pluripotency of EpSCs. Results also showed that Nanog and β-catenin are positively involved in regulating EpSCs proliferation.

Results of the western blotting demonstrated that Nanog protein expression increased 24 and 48 h after cell differentiation but with a slight decrease 72 h after cell differentiation, while β-catenin protein expression increased stably during the differentiation process. Results of the PCR analysis during the differentiation period demonstrated that Nanog mRNA expression decreased with culture time while the β-catenin mRNA expression increased with culture time. It has been proposed that Myc-induced differentiation acts as a fail-safe device to prevent uncontrolled epidermal stem cell proliferation (19,20). Myc mRNA expression was also increased during the cell differentiation process. From the results we get to know that Nanog may be negatively involved in the differentiation process of EpSCs, while β-catenin may be positively involved in the differentiation process of EpSCs.

In summary, our study showed that both the Nanog signaling pathway and the Wnt/β-catenin signaling pathway are tightly involved in the proliferation and differentiation process of EpSCs. These two signaling pathways may be linked to each other through modulation of β-catenin. However, additional studied are needed to elucidate the interaction mechanisms between these two signaling pathways during the proliferation and differentiation processes of EpSCs.
